# Rice arbuscular mycorrhiza as a tool to study the molecular mechanisms of fungal symbiosis and a potential target to increase productivity

**DOI:** 10.1186/s12284-015-0067-0

**Published:** 2015-10-30

**Authors:** Tomomi Nakagawa, Haruko Imaizumi-Anraku

**Affiliations:** Division of Symbiotic Systems, National Institute for Basic Biology, Nishigonaka 38, Myodaiji, Okazaki, Aichi 444-8585 Japan; Division of Biological Science, Graduate School of Science, Nagoya University, Nagoya, Aichi 464-8602 Japan; Division of Plant Sicences, National Institute of Agrobiological Sciences, 2-1-2 Kannon-dai, Tsukuba, Ibaraki 305-8602 Japan

**Keywords:** Arbuscular mycorrhizal (AM) symbiosis, Phosphate transporter, Common symbiosis pathway (CSP), Mycorrhizal (Myc) factors, Lysine motif (LysM) receptor-like kinases, Strigolactones (SLs)

## Abstract

Rice (*Oryza sativa* L.) is a monocot model crop for cereal molecular biology. Following the emergence of molecular genetics of arbuscular mycorrhizal (AM) symbiosis in model legumes in the 1990s, studies on rice genetic resources have considerably contributed to our understanding of the molecular mechanisms and evolution of root intracellular symbioses.

In this review, we trace the history of these studies and suggest the potential utility of AM symbiosis for improvement in rice productivity.

## Introduction

**Fig. 1:**
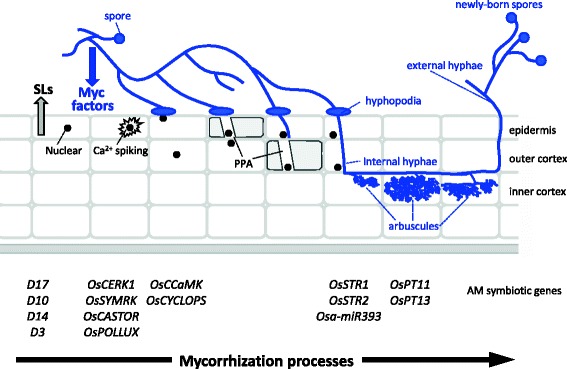
Schematic view of mycorrhization and rice genes directly involved at each stage. SLs derived from host roots induce hyphal branching of AM fungi. At the same time, Myc factors secreted from AM fungi induce symbiotic Ca^2+^ spiking. At the tips of branched hyphae, hyphopodia are formed on the epidermis of host roots. Immediately beneath the hyphopodia, the pre-penetration apparatus (PPA) develops to guide hyphal penetration. In the inner cortex, arbuscules develop and act as a nutrient exchanger between the host plants and AM fungi

Arbuscular mycorrhizal (AM) symbiosis is an ancient endosymbiosis that originated more than 400 million years ago (Remy et al. 1994). Most land plants engage in a cooperative relationship with obligate biotrophic fungi of the phylum Glomeromycota. Strigolactones (SLs) secreted by plant roots into the rhizosphere act as branching factors for AM fungal hyphae (Akiyama et al. 2005). Fungal exudates induce symbiotic Ca^2+^ spiking in the host cells, which is crucial for AM fungal infection. AM fungi penetrate the root epidermis through hyphopodia. The pre-penetration apparatus (PPA), an intracellular pre-infection structure, is formed in the host cells to determine the penetration path of fungal hypha from the epidermis to the cortex (Genre et al. 2005, Oldroyd 2013, Recorbet et al. 2013). A highly branched hyphal structure, an arbuscule, develops in the cortical cells and serves for nutrient exchange between the host plant (which provides photosynthates) and the AM fungus (which provides mainly phosphate along with other nutrients). External hyphae growing from the mycorrhizal roots allow host plants to assimilate phosphate from outside the root zone (Fig. 1) and therefore to survive in phosphorus-deficient soils.

## Review

### Identification of AM-specific phosphate transporters

Identification of AM-specific phosphate transporters was the starting point of molecular genetic research on AM symbiosis in rice. Using genome-wide data for rice (*Oryza sativa* L.), Paszkowski and colleagues have identified 13 phosphate transporter genes (*PT*) and determined that *OsPT11* is specific to AM symbiosis (Paszkowski et al. 2002). The OsPT11–GFP fusion protein is specifically localized in the periarbuscular membrane surrounding young and mature arbuscules, where active phosphate transfer seems to occur (Kobae and Hata 2010). Using *OsPT11* and *OsPT13* knockout and knockdown mutants, Yang et al. (2012) have shown that not only *OsPT11* but also *OsPT13* is involved in AM symbiosis development. Although OsPT13 is essential for AM symbiosis, symbiotic phosphate uptake is independent of OsPT13. These results indicate functional specialization: OsPT11 may be responsible for both AM development and symbiotic phosphate uptake, whereas OsPT13 may act as a sensor to detect the phosphate level appropriate for arbuscule development (Yang et al., 2012).

### Rice as a model monocot plant to study the evolution of root nodule and AM symbioses

Since the emergence of two legume models during 1990’s, *Lotus japonicus* and *Medicago truncatula*, the door for analysis of molecular mechanisms of root symbioses has been opened. Legumes have two mutualistic symbiosis systems, root nodule (RN) and AM symbiosis.

Before the molecular genetics era in legume symbiosis, the presence of host plant genes governing both RN and AM symbioses has been suggested (Resenders et al. 2001). In line with this, over half of non-nodulation mutants of *L. japonicus* and *M. truncatula* also showed non-mycorrhization phenotypes (Kouchi et al. 2010, Oldroyd 2013). A set of genes shared by both symbioses was assumed to encode key components of the “common symbiosis pathway” (CSP) that regulates intracellular infection with rhizobial bacteria and AM fungi. Extensive studies since the 2000s have revealed that these CSP components function as activators of Ca^2+^ spiking (*LjSYMRK/MtDMI2*, *LjCASTOR*, *LjPOLLUX/MtDMI1*, *LjNUP85*, *LjNUP133*, and *LjNENA*), a decoder of symbiotic Ca^2+^ signals (*LjCCaMK/MtDMI3*), and activators of downstream symbiotic signaling pathways (*LjCYCLOPS/MtIPD3*, *MtVapyrin*, *LjNSP1*, *MtNSP2*, and *LjCerberus*, reviewed by Kouchi et al. 2010 and Oldroyd 2013).

AM symbiosis is formed between the majorities of plant species, while RN symbiosis is limited to a clade within Eurosid I. Thus, it has been proposed that RN symbiosis has originated from AM symbiosis by using basic CSP and a newly acquired set of genes for the RN specific pathway (Kistner and Parniske 2002). To examine this hypothesis, comparative analysis of orthologous genes between legumes and non-legumes became essential. Among the non-leguminous plants, rice is the most suitable monocot model plant, because its genome databases and mutant resources have already been established (Miyao et al. 2003, Hirochika et al. 2004). Zhu et al. (2006) reported the presence of putative rice orthologs of the legume genes *NFR1*, *NFR5*, *SYMRK/DMI2*, *CASTOR*, *POLLUX/DMI1*, *DMI3*, *MtNSP1*, and *MtNSP2*. Studies on knockout rice mutant lines showed that the *OsCSP* genes, *OsCASTOR*, *OsPOLLUX*, *OsCCaMK*, and *OsCYCLOPS*, are directly involved in AM symbiosis (see references in Table 1).

**Table 1:** List of rice genes whose involvement for AM and/or RN symbioses have been proven by rice mutant analysis and/or heterologous complementation analysis with legumes

Gene	Gene ID	Protein annotation	Mycorrhization mutant lines	Mycorrhization phenotype	RN rescue by Os ortholog	AM rescue by Os ortholog	Ref.
OsCERK1	Os08g0538300	LysM receptor kinase	*Oscerk1*-KO (null mutants^*2^)	*Oscerk1*-KO: delayed arbuscule formation	*Ljnfr1*: Nod^+^Fix^+*8^	n/a^*9^	Miyata et al. (2014)
			OsCERK1-RNAi	OsCERK1-RNAi:Myc-			Zhang et al. (2015)
OsSYMRK	Os07g0568100	LRR protein kinase	no hit^*3^	-	*Ljsymrk*: Nod^+^Fix^−^	*Ljsymrk*: Myc^+^	Markmann et al. (2008)
OsCASTOR	Os03g0843600	Cation ion channel	*Oscastor-1* (1B-08643^*4^)	Myc^−^	*Ljcastor*: Nod^+^Fix^+^	*Ljcastor*:Myc^+^	Banba et al. (2008)
							Gutjahr et al. (2008)
							Chen et al. (2009)
OsPOLLUX	Os01g0870100	Cation ion channel	*Ospollux-1* (1C-03411^*4^)	Myc^−^	*Ljpollux*: Nod^±^Fix^−^	*Ljpollux*: Myc^±^	Banba et al. (2008)
			*Ospollux-2* (NC6423^*5^)		*Mtdmi1*: Nod^+^Fix^−^	*Mtdmi1*: Myc^+^	Gutjahr et al. (2008)
			*Ospollux-3* (ND5050^*5^)				Chen et al. (2009)
OsCCaMK/OsDMI3	Os05g0489900	Ca^2+^/CaM-dependent protein kinase	*Osccamk-1* (NE1115^*5^)	Myc^−^	*Ljccamk*: Nod^+^Fix^+^/Spn^+^	*Ljccamk*: Myc^+^	Godfroy et al. (2006)
			*Osccamk-2* (NF8513^*5^) OsDMI3-i (RNAi)		*Mtdmi3*: Nod^+^Fix^−^	*Mtdmi3*: Myc^+^	Chen et al. (2007)
							Banba et al. (2008)
							Gutjahr et al. (2008)
OsCYCLOPS/OsIPD3	Os06g0115600	interacting protein of CCaMK	*Oscyclops-1* (NG0782^*5^)	Myc^−^	*Ljcyclops*: Nod^+^Fix^+^	*Ljcyclops*:Myc^+^	Yano et al. (2008)
			*Oscyclops-2* (NC2415^*5^)				Gutjahr et al. (2008)
			*Oscyclops-3* (NC2713^*5^)				Chen et al. (2008)
			ND5032^*5^				
			NC0263^*5^				
			NC2794^*5^				
OsNSP1	Os03g0408600	GRAS TF	no hit^*3^	-	*Ljnsp1*: Nod^+^Fix^+^	-	Yokota et al. (2010)
OsNSP2	Os03g0263300	GRAS TF	no hit^*3^	-	*Ljnsp2*: Nod^+^Fix^+^	n/a^*9^	Yokota et al. (2010)
OsPT11	Os01g0657100	Phosphate transporter	*Ospt11-1* ^*4^	Low mycorrhizaion (small arbuscules)	-	-	Yang et al. (2012)
			*Ospt11R1* (RNAi)				
OsPT13	Os04g0186800	Phosphate transporter	*Ospt13-1* ^*7^	Low mycorrhizaion (small arbuscules)	-	-	Yang et al. (2012)
			*Ospt13R1* (RNAi)				
OsSTR1	Os09g0401100	half-size ABC transporter	*str1-1* (1C-04850^*4^)	Small and stunted arbuscules in cortex	-	-	Gutjahr et al. (2012)
			*str1-2* (CL522472^*6^)				
OsSTR2	Os07g0191600	half-size ABC transporter	*str-2-1* (RdSpm2654D^*7^)	Small and stunted arbuscules in cortex	-	-	Gutjahr et al. (2012)
D17	Os04g0550600	CCD7/SL biosynthesis	*d17-1*	Low mycorrhization	-	-	Umehara et al. (2008)^*10^
							Gutjahr et al. (2012)
							Yoshida et al. (2012)
D10	Os01g0746400	CCD8/SL biosynthesis	*d10-1*	Low mycorrhization	-	-	Umehara et al. (2008) ^*10^
			*d10-2*				
							Gutjahr et al. (2012)
D14	Os03g0203200	α/β-fold hydorolase/SL signaling	*d14-1*	High mycorrhization	-	-	Ishikawa et al. (2005)^*10^
							Yoshida et al. (2012)
D3	Os06g0154200	F-box Leu-rich repeat protein/SL signaling	*d3-1*	Myc^−^	-	-	Yoshida et al. (2012) ^*10^
			*d3-2*				
			*d3-3*				
osa-miR393a	MIMAT0000957^*1^	microRNA down-regulates auxin receptor genes	ox-Os-miR393	Low mycorrhization	-	-	Etemadi et al. (2014)

*1: miRBase: the microRNA database (http://www.mirbase.org/index.shtml)

*2: gene targeting lines lacking initiation codon

*3: no hit in Tos17 open line

*4: mutant lines derived from POSTECH (http://cbi.khu.ac.kr/RISD_DB.html)

*5: mutant lines derived from NIAS Tos17 rice mutant panel (https://tos.nias.affrc.go.jp/)

*6: mutant lines derived from FST-Genoplante (http://oryzatagline.cirad.fr/)

*7: Rice Transposon Flanking Sequence Tag Database (http://sundarlab.ucdavis.edu/rice/blast/blast.html)

*8: tested by a chimeric gene consisting of a ectodomain of LjNFR1 and a intracellular domain of OsCERK1

*9: these mutants show normal mycorrhization phenotype in *L. japonicus*

*10: articles in which information of mutant lines are described.

Nod^−^: non-nodulation

Nod^+^Fix^−^: nodulation + bacterial entry-

Nod^±^Fix^−^: occasional rescue of nodulation + bacterial entry-

Nod^+^Fix^+^: nodulation + nitrogen fixation+

Spn^+^: spontaneous nodulation+

Myc^−^: no cortex invasion and no arbuscule formation

Myc^±^: occasional rescue of mycorrhization+

Myc^+^: mycorrhization+

Heterologous complementation of legume symbiotic mutants with rice orthologs revealed that almost all rice genes examined rescued mycorrhization-defective phenotypes of corresponding mutants of *L. japonicus* or *M. truncatula* (Table 1). These results show functional conservation of these genes between rice and legumes in the regulation of AM symbiosis.

*OsSYMRK*, which encodes a symbiotic leucine-rich repeat (LRR) receptor kinase, failed to rescue bacterial endosymbiosis of the nodulation-defective *Ljsymrk* mutant (Markmann et al. 2008). In contrast to the other *OsCSP* genes, which have similar domain structures as those of legumes (Banba et al., 2008, Gutjahr et al., 2008, Yano et al., 2008), a comparison of structure among *SYMRK* orthologs showed the presence of additional domains at the N-terminus of LjSYMRK. Thus, ‘full-version’ LjSYMRK regulates both RN and AM symbioses, whereas ‘short-version’ OsSYMRK regulates AM symbiosis only, suggesting that stepwise domain acquisition by SYMRK has contributed to the evolution of RN symbiosis (Markmann et al. 2008).

In *L. japonicus*, CASTOR and POLLUX are twin cation channels that function non-redundantly (Imaizumi-Anraku et al. 2005, Venkateshwaran et al. 2012). Neither rhizobial nor mycorrhizal symbioses are complemented in the roots of *Ljpollux* mutant expressing *OsPOLLUX* (*OsPOLLUX*/*Ljpollux*), while both rhizobial and mycorrhizal symbioses occur in the roots of *OsCASTOR*/*Ljcastor* (Banba et al. 2008). In this view, incomplete rescue of *Ljpollux* by *OsPOLLUX* may be caused by incompatibility between LjCASTOR and OsPOLLUX in *Ljpollux* mutant, conversely, heterologous combination of OsCASTOR and LjPOLLUX may be sufficient to rescue *Ljcastor* mutant. In *M. truncatula*, DMI1, the ortholog of POLLUX, acts in the absence of CASTOR, because a serine-to-alanine substitution in its filter region, a highly conserved ADS/AGNHA amino acid residues in the 4^th^ transemembrane domain, confers the integrated function of CASTOR and POLLUX (Venkateshwaran et al., 2012). In the *Mtdmi1* mutant, *OsPOLLUX* cannot rescue rhizobial endosymbiosis, probably because *OsPOLLUX* retains the Ser residue in the filter region (Imaizumi-Anraku et al. 2005, Chen et al. 2009).

*OsCCaMK* and *OsCYCLOPS* rescue nodulation-defective phenotypes of the corresponding mutants of *M. truncatula* and/or *L. japonicus* (Godfroy et al. 2006, Chen et al. 2007, Banba et al. 2008, Yano et al. 2008, Chen et al. 2008). *CCaMK/DMI3* encodes a Ca^2+^, CaM-dependent protein kinase, which consists of kinase, CaM-binding (CaMBD), and EF-hand domains. CCaMK is a key player in RN and AM symbioses and acts as a decoder of symbiotic Ca^2+^ signals. Functional analysis of CCaMK revealed the dispensability of its CaMBD and EF-hand domains for AM symbiosis, because the mutants with deleted CaMBD and EF hand domains are able to accommodate AM fungi (Shimoda et al., 2012, Takeda et al. 2012). On the other hand, full-length CCaMK is indispensable for the infection of rhizobial bacteria (Shimoda et al. 2012). These results raise the possibilities that CCaMK plays a role in sorting the signals derived from AM or RN symbioses and that RN symbiosis requires more complex regulation of CCaMK through CaMBD/EF hand domains (Takeda et al. 2012). The domain structure of CCaMK orthologs from non-leguminous mycorrhizal plants is similar to that of legumes (Yang et al. 2011). In abscisic acid signaling, OsCCaMK induces antioxidant defense (Shi et al. 2012, 2014), which implies its yet unidentified roles in non-leguminous plants. CYCLOPS, a phosphorylation target of CCaMK, plays a role in transactivation of the *NIN* gene in the RN–specific pathway (Singh et al. 2014), whereas the downstream target of CYCLOPS in AM symbiosis remains unknown.

These complementation studies discussed above suggest that the CSP genes, except *SYMRK*, have remained unchanged during the evolution of RN symbiosis and constituted the molecular basis for the symbiotic signaling.

### Is CSP also involved in responses to infecting microorganisms in rice?

There is a myriad of microorganisms in the rhizosphere. Do they interact with plant roots via CSP? The rice blast fungus *Magnaporthe oryzae* infects the roots of *Oscastor-1* and *Osccamk-2* mutants similarly to those of wild type, indicating that the OsCSP genes are not required for this infection (Marcel et al. 2010). Endophytic colonization of the rice mutants *Oscastor-1*, *Osccamk-2*, and *Oscyclops* (NC2794) by rhizobia also appears not to be affected under laboratory conditions (Chen and Zhu 2013). However, the *OsCCaMK* genotype affects the diversity of the bacterial community in rice roots in paddy and upland fields (Ikeda et al. 2011). Minamisawa and colleagues showed that the abundance of *Alphaproteobacteria* (*Sphingomonadales* and *Rhizobiales*), which are ubiquitous in the environment, was drastically decreased on the *Osccamk-1* roots in comparison with wild-type roots (Ikeda et al. 2011). In the paddy fields under low nitrogen conditions, the methanotrophic community (mainly *Methylomonas* and *Methylomicrobium* in type I methanotrophs) was less diverse in the *Osccamk-1* roots than in the wild-type roots; the enhanced CH_4_ flux of the *Osccamk-1* mutant suggests a positive influence of *OsCCaMK* on CH_4_ oxidation (Bao et al. 2014).

### Choice between symbiosis and defense response: two faces of OsCERK1

In RN symbiosis, CSP is activated upon recognition of rhizobial symbiotic signal molecules, Nod factors, by host lysine motif (LysM) receptor-like kinases, NFR1/LYK3 and NFR5/NFP. The Nod factors are diversely modified derivatives of lipochitooligosaccharides (LCOs) and are also called Nod-LCOs. Similarly to rhizobia, AM fungi secrete diffusible signal molecules (Kosuta et al. 2003). One type of the mycorrhizal (Myc) factors, Myc-LCOs, are structurally similar to Nod-LCOs, but have a simpler structure (Maillet et al. 2011), implying that the receptors for AM fungal signals are also similar to those for Nod-LCO receptors. This suggestion is supported by the fact that a single copy of an NFR5/NFP homolog participates in both RN and AM symbioses in *Parasponia*, the only known non-legume plant that establishes rhizobial symbiosis (Op den Camp et al. 2011).

Myc-LCOs improve mycorrhization (Maillet et al. 2011). In *M. truncatula*, they induce the expression of a number of genes, which partially overlap with but are largely distinct from the genes induced by Nod-LCOs (Czaja et al. 2012). Curiously, most of these responses in *M. truncatula* require NFP (Maillet et al. 2011, Czaja et al. 2012), although the mycorrhization phenotype of *Mtnfp* appears not to be affected. These perplexing results might indicate the different dependence of AM symbiosis on the Myc-LCO/NFP system among plant species. It is noteworthy that NFR5/NFP homologs are found in a wide range of mycorrhizal plants (Zhu et al. 2006, Zhang et al. 2007), suggesting their importance.

Homologs of NFR1/LYK3 are also conserved among plants (Zhu et al. 2006, Zhang et al. 2007, De Mita et al. 2014). OsCERK1, the closest rice homolog of NFR1/LYK3, recognizes chitin or peptidoglycan and triggers plant immunity responses (Miya et al. 2007, Shimizu et al. 2010, Willmann et al. 2011). Although their physiological roles—acceptance or rejection of infecting microbes—are seemingly opposite, these genes may be descendants of a common ancestral gene (Nakagawa et al. 2011, Oldroyd 2013, Miyata et al. 2014). This view is supported by transient induction of a number of defense genes by Nod-LCOs in an NFR1-dependent manner in *L. japonicus* (Nakagawa et al. 2011) and by the findings that co-expression of either LjNFR1 with LjNFR5 or MtLYK3 with MtNFP causes cell death in *Nicotiana benthamiana* (Madsen et al. 2011, Pietraszewska-Bogiel et al. 2013). Therefore, NFR1/LYK3 also bears some traits related to defense responses, a feature reminiscent of CERK1.

The ectodomains of NFR1 and CERK1 distinguish between Nod-LCOs and chitin oligomers (Broghammer et al. 2012, Wang et al. 2014), and their intracellular kinase domains play crucial roles in triggering nodulation or immunity responses, respectively (Petutschnig et al. 2010, Madsen et al. 2011). Despite the contrasting functions of the two kinases, chimeric constructs consisting of the ectodomain of LjNFR1 and the kinase domain of OsCERK1 rescue the nodulation defect of the *Ljnfr1* mutant (Nakagawa et al. 2011, Miyata et al. 2014), suggesting the ability of OsCERK1 to regulate symbiotic interactions. Indeed, the *Oscerk1* null mutants (Kouzai et al. 2014a) are impaired in both chitin-triggered immunity and AM symbiosis (Miyata et al. 2014). Yet, these two functions are not necessarily coupled because the *Oscebip* mutant (Kouzai et al. 2014b) has defective chitin-triggered immunity but normal AM symbiosis (Miyata et al. 2014). In the *oscerk1* null mutants, AM fungal infection is initially blocked at the root surface but prolonged cultivation eventually allows penetration of fungal hyphae into the root cortex and arbuscular formation (Miyata et al. 2014). In contrast, OsCERK1-knockdown lines clearly show an impaired mycorrhization phenotype (Zhang et al. 2015) even at the time point corresponding to "prolonged cultivation" in *oscerk1* null mutants in the study by Miyata et al. (2014). Although the mycorrhizal conditions differed in the two studies, different phenotypes of the *oscerk1* null mutants and OsCERK1-knockdown lines might imply the existence of a functionally redundant gene. The bifunctionality of OsCERK1 raises a question of how the same receptor selectively triggers the opposite physiological responses. In response to microorganisms, OsCERK1 appears to recruit appropriate receptor partners to form symbiotic or defensive receptor complexes by which the initial interactions are determined.

### Reverse genetic approaches of rice provide new insights into AM symbiosis

Reverse genetic approaches have identified novel AM symbiosis genes in rice. Genome-wide microarray analysis of mycorrhized rice roots revealed 224 genes the expression of which was altered in response to mycorrhization (Güimil et al. 2005). These *OsAM* genes were used for molecular phenotyping of the *Oscsp* mutants, which resulted in identification of an alternative signaling pathway independent of CSP (Gutjahr et al. 2008). Furthermore, 76 of *OsAM* genes showed similar expression patterns of mono- and dicotyledonous plants, shedding light on a conserved molecular mechanism governing AM symbiosis (Güimil et al. 2005).

The expression of two half-size ABC transporters, STR1 and STR2, is enhanced during AM symbiosis, and these transporters are directly involved in arbuscule development (Gutjahr et al. 2012).

Two markers, OsPT11–GFP, localized in the periarbuscular membrane (Kobae and Hata 2010) and GFP–AM42, localized in the arbuscule, enable observation of arbuscule development (Kobae and Fujiwara 2014). Long-term live imaging of transgenic OsPT11–GFP and GFP–AM42 rice roots revealed a short life-span of arbuscules and repetitive *de novo* colonization of rice roots by AM fungi.

Plant hormones, e.g., abscisic acid, gibberellin, auxin, and SLs, are involved in AM symbiosis (Foo et al. 2013). In rice, the overexpression of *osmiR393*, a microRNA that targets several auxin receptors, results in down-regulation of auxin receptor genes and hampers arbuscule development (Etemadi et al. 2014).

### Relationship between strigolactone biosynthesis and mycorrhization

Identification of SLs as branching factors (Akiyama et al. 2005) has led to a breakthrough in understanding of the molecular mechanisms of the pre-contact stage of AM symbiosis. SLs also act as plant hormones to regulate shoot branching (Domagalska and Leyser 2011). In rice, the causative genes of the increased-tiller-outgrowth mutants, *d17*, *d10*, *d27*, *d14* and *d3* have been identified as SL-biosynthesis (*D17*, *D10* and *D27*) or SL-signaling (*D14* and *D3*) genes. In *d10* and *d17* mutants, the rates of colonization by AM fungi are decreased, whereas arbuscule structures are not affected (Gutjahr et al. 2012), indicating that SLs are dispensable for arbuscule development. Because SLs are undetectable in root exudates of these mutants (Umehara et al. 2008), their mycorrhization phenotypes are likely to be associated with incomplete induction of hyphal branching, which may determine the lower frequency of AM fungal infection.

The crosstalk between the SL synthesis pathway and the GRAS transcription factors NSP1 and NSP2 has been shown in rice. A decreased expression of *D27* was detected in the *Osnsp1*/*Osnsp2* double knockdown lines (Liu et al. 2011) and in the *nsp1*, *nsp2*, and *nsp1*/ *nsp2* background of *M. truncatula* and *L. japonicus* (Liu et al. 2011, Takeda et al. 2013, Nagae et al. 2014). However, the colonization rate of the *Ljnsp1* mutant was not restored by application of SL analog, GR24, suggesting that the attenuation of the SL biosynthesis pathway is not a major determinant of the mycorrhization defect of this mutant (Takeda et al. 2013).

The *d3* and *d14* mutants are SL-insensitive and synthesize more SLs and induce highly branched hyphae around their roots in comparison with wild type (Umehara et al. 2008, Yoshida et al. 2012). As expected, *d14* shows a high-mycorrhization phenotype. However, the development of hyphopodia on *d3* roots is arrested (Yoshida et al. 2012) and the mode of action of D3 at the initial penetration stage of AM fungi remains elusive.

## Conclusions and Perspective

As reviewed here, rice has made a major contribution to the elucidation of molecular mechamisms of AM symbiosis, as a model monocot plant. On the other hand, rice is one of three major cereals and has a direct impact on food supplies. Conventional agriculture largely depends on the input of phosphorus fertilizer; the output of mineral phosphorus is expected to reach a peak around 2030, and then to take a downward turn (Cordell et al. 2009). Under these circumstances, rice cultivars that need less phosphorus are an important breeding target (Kochian 2012). As a successful example of such approach, the protein kinase *Pstol1*, initially detected in cultivar Kasalath as a QTL for phosphorus-deficiency tolerance (*Pup1*), was used to confer tolerance to phosphorus deficiency in phosphorus-starvation-intolerant modern cultivars. *Pstol1* acts as an enhancer of root growth, thereby allowing host plants to absorb more phosphorus (Gamuyao et al. 2012).

In general, rice is grown in flooded fields, where mycorrhization is inhibited under anaerobic conditions, in part because of reduced spore abundance of AM fungi in comparison with dry conditions (Ilag et al. 1987). Regardless of rice varieties, colonization by AM fungi is substantially reduced under flooded conditions (Vallino et al. 2009). AM symbiosis can be used for rice cultivation under flooded conditions (Secilia and Bagyaraj 1992, 1994, Solaiman and Hirata 1997, 1998); however, rice breeding to use AM symbiosis for phosphate uptake remains out of reach.

Even though modern rice cultivars are grown under anaerobic conditions, the amount of inorganic phosphate (Pi) uptake through AM symbiosis accounts for 70 % of total Pi absorbed by rice cultivar Nipponbare under experimental condition. In addition, cultivar IR66 maintains elevated expression levels of AM marker genes under both aerobic and anaerobic field conditions, suggesting high dependency of rice Pi uptake on the mycorrhizal pathway (Yang et al. 2012). Although rice flooding has a negative impact on AM fungal colonization, AM fungi that had entered into rice roots before flooding remain viable in the roots under anaerobic conditions. Furthermore, basic functionality of AM symbiosis is not affected by flooding (Maiti et al. 2008 and Vallino et al. 2014). Therefore, rice seems to be a potential target for breeding to increase productivity through AM symbiosis.
